# Ewing sarcoma from molecular biology to the clinic

**DOI:** 10.3389/fcell.2023.1248753

**Published:** 2023-09-11

**Authors:** Maryne Dupuy, François Lamoureux, Mathilde Mullard, Anaïs Postec, Laura Regnier, Marc Baud’huin, Steven Georges, Bénédicte Brounais-Le Royer, Benjamin Ory, Françoise Rédini, Franck Verrecchia

**Affiliations:** Nantes Université, Inserm UMR 1307, CNRS UMR 6075, CRCI2NA, Université d'Angers, Nantes, France

**Keywords:** ewing sarcoma, EWS-FLI1, clinical, molecular biology, cellular biology

## Abstract

In Europe, with an incidence of 7.5 cases per million, Ewing sarcoma (ES) is the second most common primary malignant bone tumor in children, adolescents and young adults, after osteosarcoma. Since the 1980s, conventional treatment has been based on the use of neoadjuvant and adjuvant chemotherapeutic agents combined with surgical resection of the tumor when possible. These treatments have increased the patient survival rate to 70% for localized forms, which drops drastically to less than 30% when patients are resistant to chemotherapy or when pulmonary metastases are present at diagnosis. However, the lack of improvement in these survival rates over the last decades points to the urgent need for new therapies. Genetically, ES is characterized by a chromosomal translocation between a member of the FET family and a member of the ETS family. In 85% of cases, the chromosomal translocation found is (11; 22) (q24; q12), between the EWS RNA-binding protein and the FLI1 transcription factor, leading to the EWS-FLI1 fusion protein. This chimeric protein acts as an oncogenic factor playing a crucial role in the development of ES. This review provides a non-exhaustive overview of ES from a clinical and biological point of view, describing its main clinical, cellular and molecular aspects.

## 1 Introduction

Sarcomas are defined as solid tumors that develop from connective tissue. In contrast to carcinomas, which are solid tumors that develop from epithelial cells, sarcomas originate in mesenchymal cells. These sarcomas can be classified into three main groups (Sbaraglia et al., 2021).

1) Soft tissue and visceral sarcomas such as liposarcomas, fibro-myofibroblastic sarcomas, leiomyosarcomas, rhabdomyosarcomas, vascular sarcomas, gastrointestinal stromal tumors, sarcomas with bone or cartilage differentiation, malignant nerve sheath tumors, undifferentiated sarcomas.
2) Bone sarcomas such as osteosarcomas, chondrosarcomas, bone fibrosarcomas, bone angiosarcomas, bone leiomyosarcomas and undifferentiated polymorphic sarcomas.
3) Undifferentiated small round cell sarcomas of bone and soft tissue such as Ewing sarcomas.

## 2 Ewing sarcoma (ES)

### 2.1 Main clinical characteristics

#### 2.1.1 Primary bone tumors

Twenty-one percent of pediatric cancers are defined as sarcomas (Burningham et al., 2012), of which 3% are primary bone tumors (Stiller et al., 2013; Jackson et al., 2016; Aran et al., 2021). ES is thus the second most common primary malignant bone tumor in children, adolescents, and young adults, after osteosarcoma. Together, these two tumors account for around 90% of pediatric bone sarcomas (Desandes et al., 2004; Raze et al., 2021).

#### 2.1.2 General overview

ES was first described by James Ewing in 1921 as a new bone tumor called “diffuse bone endothelioma” (Ewing, 1972). Initially, the World Health Organization classification grouped ES tumors, primitive neuroectodermal tumors and Askin tumors into a single tumor group on the basis of their histological similarities and the presence of FET-ETS fusion genes (Doyle, 2014).

These tumors were distinguished from “Ewing sarcoma-like”, having morphological similarities with ES, but being characterized by other fusion genes and different clinical and pathological features (Grünewald et al., 2018). This classification was modified in 2016, notably differentiating primitive neuroectodermal tumors from bone-site or extra-bone-site ES (Louis et al., 2016).

**FIGURE 1:**
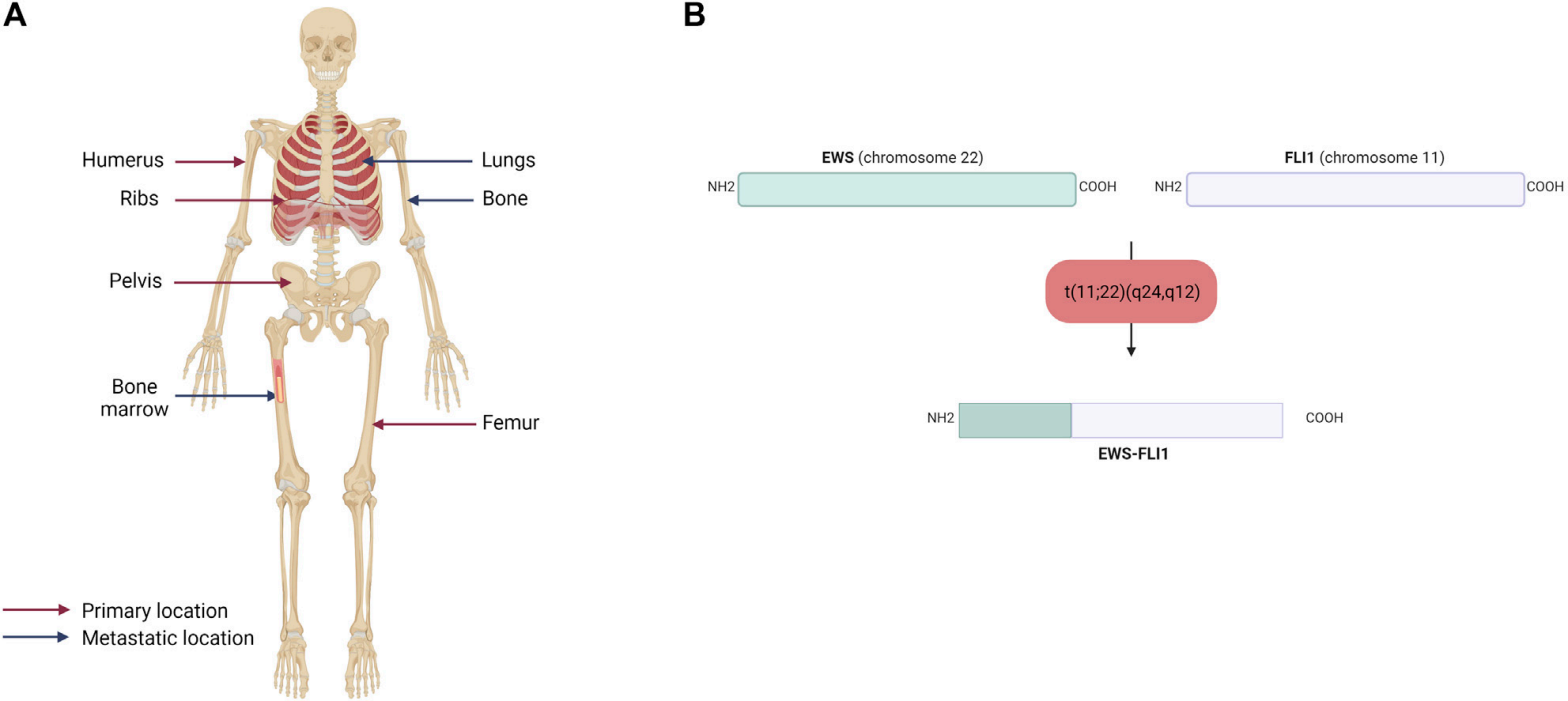
**(A)** Primary and metastatic sites of Ewing sarcoma. Ewing sarcoma mainly affects the humerus, ribs, pelvis and femur and metastasized to the lungs, bones and bone marrow **(B)** Schematic representation of the EWS-FLI1 fusion protein. The main chromosomal translocation in Ewing sarcoma occurs between EWS on the chromosome 22 and FLI1 on the chromosome 11. The resulting fusion protein displays the N-terminal domain of EWS and the C-terminal domain of FLI1.

The majority of ES are osseous, arising mainly in the pelvis and ribs, but also in the diaphysis of long bones (femur, tibia, fibula) (Figure 1A). Twenty to 30% of ES may nevertheless be extraosseous (Lynch et al., 2018; Jahanseir et al., 2020). ES can thus be located in soft tissues, such as the thoracic cavity wall or pleural cavities. ES is an aggressive tumor, including a high risk of metastases, which is a factor of poor prognosis. The percentage of patients with metastatic ES at diagnosis is estimated to be between 20% and 25%. These metastases are most often located in the lungs, bones, but also in the spinal cord in a smaller percent (Grünewald et al., 2018).

#### 2.1.3 Epidemiology

ES mainly affects children, adolescents, and young adults, with a peak incidence at 15 years of age at diagnosis (Jawad et al., 2009). In Europe, the incidence rate is 7.5 cases per year per million children aged between 10 and 19 years old (Spector et al., 2021). In addition, there are around 100 new cases every year in France. Men are slightly more affected than women, with a sex ratio of 3:2 (Hu et al., 2021). Disparities can also be observed in the distribution of ES across the population. Indeed, several studies show a very low incidence of this tumor in Asian and African populations (Jawad et al., 2009; Worch et al., 2011; Hu et al., 2021). Beyond environmental and lifestyle disparities, this has been correlated with a germline genomic variant common to European and Western populations that allows the extension of microsatellites at 6 loci (*EGR2*, Early Growth Response 2; *ADO*, 2-Aminoethanethiol Dioxygenase; *TARDBP*, TAR DNA Binding Protein; *RREB1*, Ras Responsive Element Binding Protein 1; *KIZ*, Kizuna Centrosomal Protein and *NKX2-2*, NK2 Homeobox 2) facilitating the binding of the EWS-FLI1 fusion protein (Postel-Vinay et al., 2012; Machiela et al., 2018).

#### 2.1.4 Etiology

In contrast to osteosarcoma, which is a highly heterogeneous tumor at the genetic level, ES is poor in terms of genetic abnormalities. It is characterized by a recurrent chromosomal translocation between a member of the FET family of RNA-binding proteins and a member of the ETS family of transcription factors (Table 1). The first observed chromosomal translocation, t (11; 22) (q12; 24), was described in 1984 (Aurias et al., 1984; Turc-Carel et al., 1984). In 1992, it was characterized as a chromosomal translocation between the *EWSR1* (also known as *EWS*) and *FLI1* genes, generating the EWS-FLI1 fusion protein (Delattre et al., 1992), which is characteristic of 85% of ES cases (Figure 1B). In the remaining 15% of ES cases, other fusion genes have been described, mainly involving the EWS gene with other members of the ETS family (Zucman et al., 1993; Jeon et al., 1995; Kaneko et al., 1996; Peter et al., 1997; Shing et al., 2003; Ng et al., 2007), with 10% of these cases characterized by the formation of the EWS-ERG fusion protein (Zucman et al., 1993). Nevertheless, it has been shown that patient survival does not vary according to translocation type (Le Deley et al., 2010; van Doorninck et al., 2010).

**TABLE 1:** Chromosomal translocations identified in Ewing sarcoma.

Family	Translocation	Fusion gene	Frequency
EWS-ETS	t (11; 22) (q24; q12)	EWSR1-FLI1	85%
	t (21; 22) (q22; q12)	EWSR1-ERG	10%
	t (7; 22) (p22; q12)	EWSR1-ETV1	<1%
	t (17; 22) (q12; q12)	EWSR1-ETV4	<1%
	t (2; 22) (q33; q12)	EWSR1-FEV	<1%
FUS-ETS	t (2; 16) (q35; p11)	FUS-FEV	<1%
	t (16; 21) (p11; q22)	FUS-ERG	<1%

EWS-ETS: abbreviation for EWSR1, standing for Ewing sarcoma breakpoint region 1—E-twenty six Transformation Specific. FUS-ETS: FUsed in Sarcoma—E-twenty six Transformation Specific.

On the other hand, rare other protein-coding mutations have been observed, notably in *TP53* and *STAG2* (Brohl et al., 2014; Crompton et al., 2014; Tirode et al., 2014), the latter being the most commonly mutated gene in ES (15%–21% of cases) (Brohl et al., 2014). More surprisingly, although *TP53* is mutated in more than 50% of cancers, mutations in this gene are observed in only 5%–7% of ES cases (Kovar et al., 1993; Huang et al., 2005). Other mutations have also been identified such as those affecting *EZH2* (Enhancer of Zeste 2 Polycomb Repressive Complex 2 Subunit), *BCOR* (BCL6 Corepressor), *ZMYM3* (Zinc finger MYM-type containing 3) or *CDKN2A* (Cyclin Dependent Kinase Inhibitor 2a) (Huang et al., 2005; Brownhill et al., 2007; Tirode et al., 2014).

#### 2.1.5 Diagnosis and therapeutic management

##### 2.1.5.1 Clinical diagnosis

ES is a fast-growing tumor, forming osteolytic lesions, which can lead to bone pain and sometimes to pathological fractures. The clinical picture of ES is not very distinctive, which often leads to a delay in diagnosis, ranging from several weeks to several months. The first signs that may lead to consultation are the appearance of swelling in the affected bone, associated with a slight pain that may become more pronounced at night or following physical activity. In more advanced cases, pain and the presence of a mass are accompanied by less specific symptoms, such as fever, fatigue or weight loss, which may be a sign that the tumor has become metastatic (Biswas et al., 2014; Pizzo et al., 2015).

Diagnosis includes imaging studies before confirmation with surgical biopsy and histological and molecular analyses. Imaging consists of conventional radiography, through which it is possible to see the osteolytic damage created by the tumor mass in the diaphyseal-metaphyseal bone (Riggi et al., 2021). Magnetic Resonance Imaging (MRI) is then generally prescribed, which can be combined with a tomographic examination. Thanks to better spatial resolution and contrast, this allows better visualization of calcifications and tumor extension into adjacent bone and soft tissue. A technique combining 18F-fluorodeoxyglucose (FDG), PET-scan and tomography can also be used to assess tumor regression or progression upstream of MRI (Gerth et al., 2007). It has been shown that this technique can also be used to assess the presence of spinal cord metastases, in order to avoid spinal cord puncture (Newman et al., 2013; Kopp et al., 2015; Kasalak et al., 2018).

Histological features of the sample include the observation of small, round, undifferentiated cells. These cells have a prominent nucleus and sparse cytoplasm with glycogen deposits. Classically, the marker CD99 (Cluster of Differentiation 99), a transmembrane glycoprotein, is used for the diagnosis of ES (Ambros et al., 1991). Other markers can also be used, such as CD57 (Cluster of Differentiation 57) and synaptophysin, which are neuronal markers (Riggi and Stamenkovic, 2007). However, it should be noted that CD99 is not specific to ES as it can be a marker in other round cell sarcomas and even leukemias (Baldauf et al., 2018). The definitive diagnosis relies on the identification of the fusion protein by fluorescent *in situ* hybridization (FISH) or even quantitative Polymerase Chain Reaction (qPCR) techniques.

##### 2.1.5.2 Metastatic development

**FIGURE 2:**
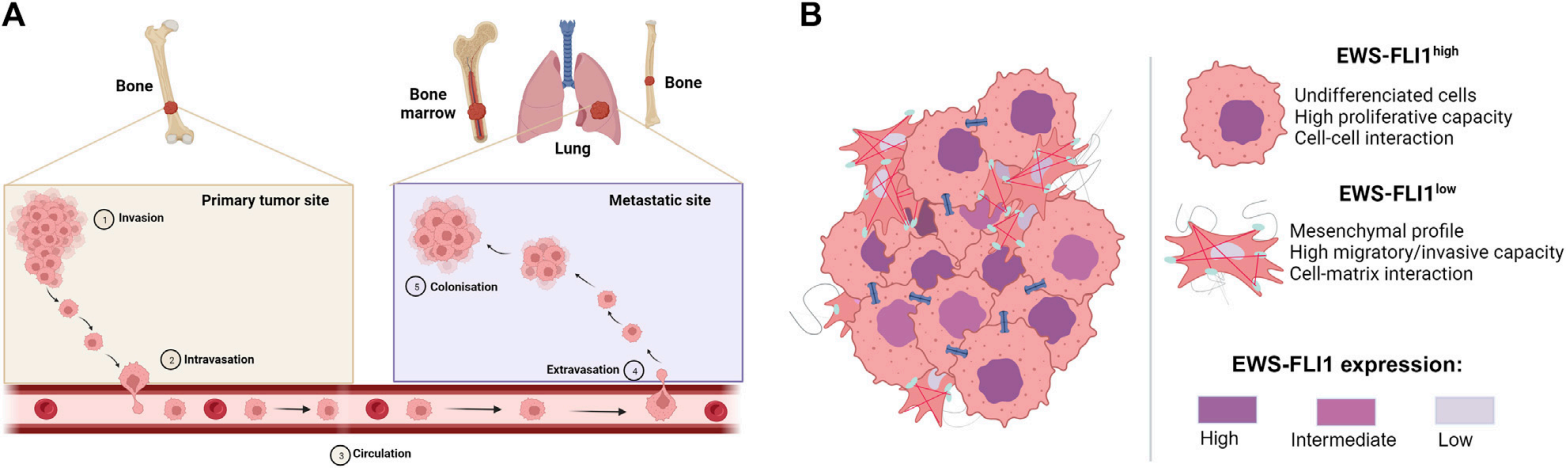
**(A)** Metastatic development of Ewing sarcoma. Tumor cells invade the primary tumor site (Invasion), before passing through the bloodstream (Intravasation) to the metastatic site, where they exit the bloodstream (Extravasation) to form metastases (Colonization) **(B)** Mechanism of proliferation and dissemination of Ewing sarcoma according to EWS-FLI1 expression (based on Franzetti et al., 2017). Cells strongly expressing EWS-FLI1 are undifferentiated, more proliferative and display more cell-cell interactions. On the other hand, cells weakly expressing EWS-FLI1 are more mesenchymal, migrate more and display more cell-matric interactions.

During the progression of ES, 20%–25% of patients may develop metastases at diagnosis (Grünewald et al., 2018; Strauss et al., 2021) to the lung (10%), bone (10%) or other sites (5%), spreading *via* the bloodstream (Casali et al., 2018) (Figure 2A).

Although many diverse processes are described to drive the metastatic development of ES, multiple studies report the crucial role of the EWS-FLI1 fusion protein in intra-tumor heterogeneity and in the ability of cells to migrate to metastatic sites (Franzetti et al., 2017; Sheffield et al., 2017; Aynaud et al., 2020). Indeed, cells with high EWS-FLI1 expression are rather undifferentiated and proliferate rapidly, whereas cells with low EWS-FLI1 expression have a more mesenchymal phenotype and are inclined to migrate and metastasize (Figure 2B). In this context, several studies have shown that EWS-FLI1 controls the metastatic potential of cells by regulating the organization of the actin cytoskeleton (Amsellem et al., 2005; Chaturvedi et al., 2012; 2014). Cells with low EWS-FLI1 expression show a loss of E-cadherin expression (Jolly et al., 2019) in favor of high expression of mesenchymal markers such as N-cadherin and Slug (Chaturvedi et al., 2012). EWS-FLI1 silencing also leads to decreased expression of cell-cell adhesion proteins such as tight junctions (CLD1, Claudin-1; OCL, Occludin) or desmosomes (DSP, Desmoplakin; PKP1, Plakophilin-1) (Franzetti et al., 2017).

##### 2.1.5.3 Prognosis

The presence of metastases at the time of diagnosis is the most important prognostic factor, significantly reducing the probability of survival from around 70% at 5 years when the tumor is localized to less than 30% for patients with metastases (Takenaka et al., 2016; Li et al., 2022). Other factors, such as the location of metastases, the patient’s age and the location of the primary tumor (Hu et al., 2021), also play a part in prognosis. Indeed, the presence of bone rather than lung metastases (Casali et al., 2018; Strauss et al., 2021), adolescents/young adults rather than children aged 0–14 (Desandes and Stark, 2016), and a primary tumor located in the pelvis, sacrum or coccyx rather than in the long bones have a worse prognosis (Hu et al., 2021).

##### 2.1.5.4 Therapeutic treatment

According to European guidelines, the therapeutic management of ES is as follows (Strauss et al., 2021): whether the tumor is localized or metastatic, the patient will undergo initial neoadjuvant chemotherapy, followed by local tumor therapy through surgical removal of the tumor and then adjuvant chemotherapy. Until recently, standard treatment in Europe combined 4 agents: Vincristine, Ifosfamide, Doxorubicin and Etoposide (VIDE) (Juergens et al., 2006; Ladenstein et al., 2010). If the primary tumor is large, or if the response to treatment is poor, the patient may be prescribed consolidation treatment with high-dose chemotherapy based on Vincristine, Actinomycin D and Ifosfamide (VAI) or Cyclophosphamide (VAC) or Busulfan and Melphalan. Because ES is a radiation-sensitive tumor (Gaspar et al., 2015), surgical resection can be supplemented with postoperative radiation therapy or even replaced by radiation therapy if the tumor is inoperable because in a site difficult to reach. Nonetheless, patients who have undergone surgical excision as well as radiation therapy have a decreased risk of recurrence compared to patients who have only undergone radiation therapy, although there does not appear to be a significant effect on overall patient survival (Foulon et al., 2016).

New therapeutic strategies are being explored to improve the prognosis and survival of ES patients, which have not improved over the past 20 years.

The EuroEwing 2012 clinical trial was aimed to compare the efficacy, survival, and toxicity of European standardized chemotherapy with the protocol used in the United States from the Children’s Oncology Group (AEWS0031 clinical trial, (Womer et al., 2012; Anderton et al., 2020). Patients on this protocol receive alternating cycles of Vincristine - Doxorubicin - Cyclophosphamide and Ifosfamide - Etoposide (VDC/I.E.,) as induction chemotherapy and then alternating cycles of Ifosfamide - Etoposide and Vincristine - Cyclophosphamide (I.E.,/VC). Initial results from this clinical trial indicate that the US protocol is more effective in terms of overall survival and event-free survival, less toxic, and shorter in duration for newly diagnosed cases of ES (Brennan et al., 2020; Brennan et al., 2022). Since this study, the interval compressed V(D)C/I.E., schedule is now standard of care across Europe and the treatment backbone for the upcoming academic trials in ES. Other clinical trials aimed at improving response to chemotherapy using other chemotherapeutic agents, alone or in combination with various drugs, are currently being studied or enrolled (Supplementary Table S1). For example, the Phase II/III multi arm multi stage clinical trial for relapsed refractory ES (rEECur) was evaluating the effect of Topotecan and Cyclophosphamide (TC), Irinotecan and Temozolomide (IT), Gemcitabine and Docetaxel (GD), or high-dose Ifosfamide (IFOS) in the treatment of recurrent and refractory ES (RR-ES) (ISRCTN36453794). The first results showed that IFOS is more effective in prolonging survival than TC, having previously overcoming GD and IT, and should be considered as a control arm in future randomized phase II/III studies in RR-ES if combination with IFOS makes sense. This trial is currently recruiting patients to IFOS and Cyclophosphamide/Etoposide regimens, and an additional arm with a molecularly targeted agent is planned, such as Multi-Tyrosine Kinase Inhibitors (MTKI) (McCabe et al., 2022).

### 2.2 Main cellular characteristics: cellular origin of ES

The origin of ES has been debated for several decades with two main theories as to the cell of origin, neural crest stem cells or mesenchymal stem cells (Kovar, 2010; Lin et al., 2011; Tu et al., 2017).

#### 2.2.1 Neural crest stem cells

Neural crest stem cells are multipotent stem cells contributing, for example, to the precursors of Schwann cells or cells of the peripheral nervous system. Various studies have shown that ES express markers of neural crest stem cells, such as CD57 (Cluster of Differentiation 57), ENO2 (Neuron-specific Enolase), S-100 or genes of the Notch signaling pathway (Franchi et al., 2001; Baliko et al., 2007; Wahl et al., 2010). Neurosecretory granules, have also been observed in ES by electron microscopy (Suh et al., 2002). Furthermore, it has been shown that ES cell lines can differentiate into neurons after specific treatments inducing neuronal differentiation (Cavazzana et al., 1987). In support of a neuroectodermal origin of ES, various studies have also demonstrated a genomic expression profile of this tumor similar to neural crest stem cells, in the presence of EWS-FLI1 (Staege et al., 2004; von Levetzow et al., 2011). However, as EWS-FLI1 is able to induce a neural crest-like phenotype and upregulates genes associated with primitive neuronal differentiation (Teitell et al., 1999; Hu-Lieskovan et al., 2005), doubts remain as to the cellular origin of ES. Indeed, the neuroectodermal characteristics of ES could be the result of EWS-FLI1 expression and not a reflection of the intrinsic properties of the cell of origin.

#### 2.2.2 Mesenchymal stem cells (MSC)

MSCs are multipotent, self-renewing stem cells derived in particular from bone marrow, able to differentiate into osteoblasts, adipocytes, chondrocytes or myocytes.

In this context, it has been shown that overexpression of EWS-FLI1 in murine MSCs leads to their transformation and the formation of sarcoma once implanted *in vivo*, with characteristics (CD99 expression) and morphology similar to those of ES (Torchia et al., 2003; Castillero-Trejo et al., 2005; Riggi et al., 2005). Expression of EWS-FLI1 in human MSCs stimulates expression of genes involved in neuronal differentiation, but is not sufficient to induce a tumor *in vivo*, in contrast to experiments with murine MSCs (Riggi et al., 2008). In addition, a study of transcriptomic profiles showed that ES cells deficient in EWS-FLI1 displayed mesenchymal characteristics (Tirode et al., 2007). In a recent study, EWS-FLI1-expressing cells were generated from “normal, non-cancerous” MSCs derived from a patient with Ewing sarcoma (Sole et al., 2021). These cells display morphological, transcriptomic and epigenetic characteristics similar to those of ES, suggesting that ES can be derived from bone marrow-derived MSCs.

### 2.3 Main molecular characteristics

#### 2.3.1 The FET protein family

Located on chromosomes 16, 22 and 17 respectively, the *FUS* (Fused In Sarcoma or *TLS*, for Translocated in Liposarcoma), *EWSR1* (Ewing sarcoma breakpoint region 1 also known as *EWS*) and *TAF15* (TATA-binding protein-associated factor 15, also known as *TAF2N*) genes belong to the FET family of RNA-binding proteins. Structurally, the *FUS*, *EWS* and *TAF15* genes are very similar, with several common domains (Tan and Manley, 2009; Schwartz et al., 2015).

- An N-terminal domain characterized by a disordered, prion-like structure, due to the presence of Serine - Tyrosine - Glycine - Glutamine (SYGQ) repeats. Its composition suggests its involvement in protein-protein interactions,
- A central Recognition RNA Motif (RRM),
- Arginine-glycine-glycine (RGG)-rich domains, also involved in protein self-assembly and RNA binding,
- A Zinc-Finger Domain, with the same role as the RGG domains in protein-RNA binding.

Due to their structure, the ubiquitously expressed genes that compose the FET protein family are involved in a wide variety of processes, such as transcription, post-transcriptional regulation and DNA damage repair (Tan and Manley, 2009; Wang et al., 2013; Schwartz et al., 2015). These proteins can affect the transcription of target genes through direct interactions with regulators or transcription factors, such as RNA polymerase II, CBP (CREB-Binding Protein)/p300, TFIID (Transcription Factor II D) or Sp1 (Bertolotti et al., 1996; Hallier et al., 1998; Wang et al., 2008; Tan and Manley, 2009; Hoell et al., 2011; Schwartz et al., 2012).

#### 2.3.2 The ETS protein family

Friend Leukemia Integration 1 (*FLI1*) is one of 26 genes in the E-twenty-six Transformation-Specific (ETS) protein family. The ETS proteins are divided into 12 subfamilies, including the ERG subfamily, which comprises 3 proteins: ERG (ETS-Related Gene), FLI1 and FEV (Fifth Ewing Variant). All proteins in this ETS family have a DNA-binding domain (ETS domain), binding a purine-rich motif (GGA [A/T]) (Karim et al., 1990; Nye et al., 1992). More specifically, FLI1 displays 4 distinct functional domains, including a 5′ETS domain, a specific FLI1 region (FLS, FLI1 Specific domain), a second 3′ETS domain, responsible for DNA binding, and a C-terminal Transcriptional Activation domain (CTA) (Wenge et al., 2015).

These proteins are known to be involved in various biological processes, such as embryonic development, vasculogenesis, angiogenesis and hematopoiesis (Oikawa and Yamada, 2003; Schober et al., 2005). In addition, these transcription factors are regularly studied for their role as oncogenic transcription activators, the latter being notably implicated in chromosomal translocation mechanisms in various types of cancer (Sizemore et al., 2017). Examples include EWS-FLI1 in ES (Delattre et al., 1992) and TMPRSS2-ERG in prostate cancer (Tomlins et al., 2005). Finally, some studies demonstrate a role for ETS proteins other than that of transcription factor. Indeed, they could be involved in post-transcriptional processes, ERG having been shown to play a role in mRNA degradation (Rambout et al., 2016).

#### 2.3.3 The EWS-FLI1 fusion protein

##### 2.3.3.1 Chromosomal translocation

**FIGURE 3:**
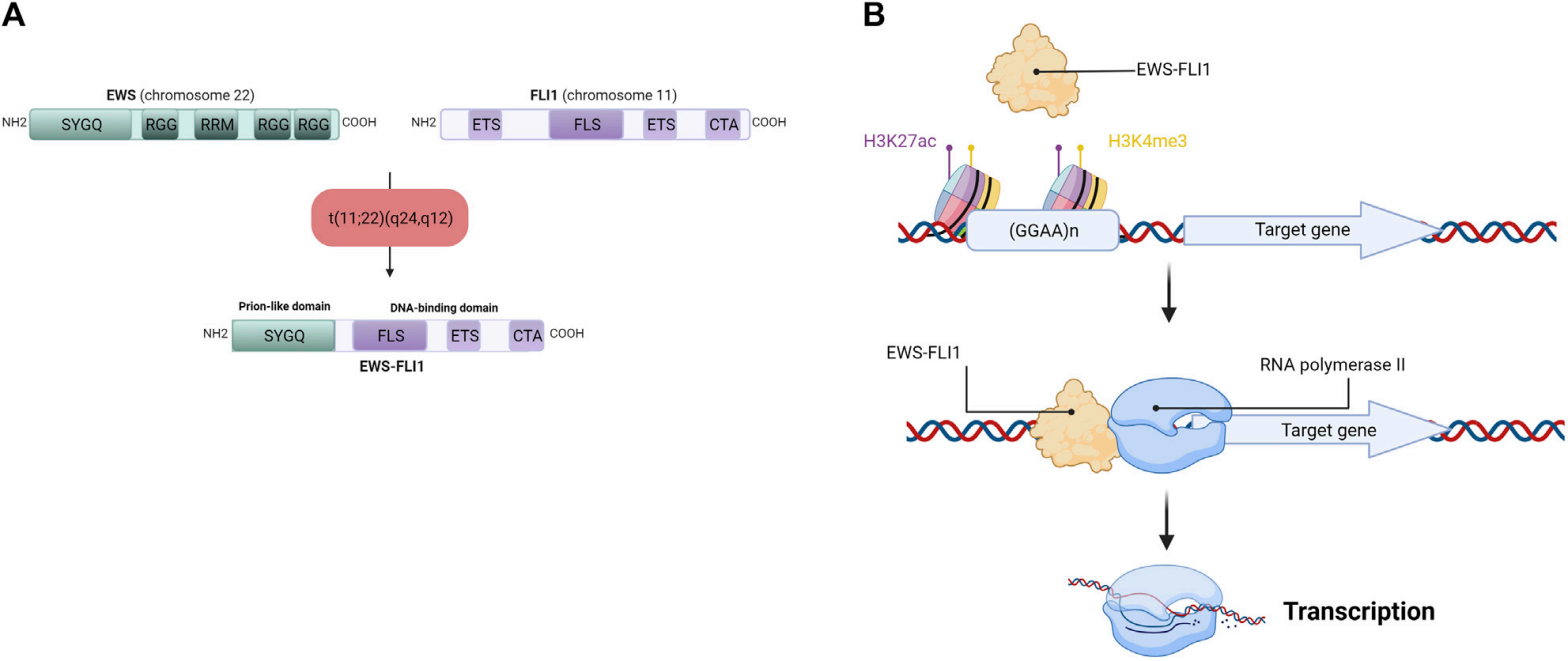
**(A)** Detailed schematic representation of the EWS-FLI1 fusion protein. EWS has Serine—Tyrosine—Glycine—Glutamine rich domain (SYGQ), Arginine—Glycine—Glycine rich domain (RGG) and a RNA Recognition Motif (RRM). FLI1 has an E26 Transformation-Specific domain (ETS), a FLI1-Specific Region (FLS) and a Carboxy-terminal Transcriptional Activation domain (CTA). After the chromosomal translocation, the EWS-FLI1 fusion protein display the SYGQ domain from EWS and the FLS, ETS and CTA domains from FLI1 **(B)** EWS-FLI1 binding at GGAA microsatellites near its target gene promoter. EWS-FLI1 is able to bind GGAA microsatellites repetitions in chromatin-opening regions (H3K27ac) and active promoter regions (H3K4me3), allowing the transcription of the target gene by the RNA polymerase II.

The chromosomal translocation t (11; 22) (q24,q12) generates the *EWS-FLI1* fusion gene (under the control of the *EWS* promoter), composed of the N-terminal domain of the *EWS* gene and the C-terminal domain of *FLI1* (Figure 3A). Depending on the position of the breakpoints in the different genes, over 10 different *EWS-FLI1* transcripts have been described in the literature (Zucman et al., 1993), the majority being between exon 7 of the *EWS* gene and exon 6 of the *FLI1* gene (Delattre et al., 1992), or between exon 7 of the *EWS* gene and exon 5 of the *FLI1* gene (Delattre et al., 1992; Zucman et al., 1993). It should be noted that the reciprocal transcript (*FLI1-EWS*) also exists, but until recently was considered to be little or not expressed in ES cells (Zucman et al., 1993). Indeed, *EWS-FLI1* is expressed under the control of the *EWS* promoter, whereas *FLI1-EWS* is expressed under the control of the *FLI1* promoter, which is not very active in ES (Zucman et al., 1993). However, a recent study shows that *FLI1-EWS* expression in some ES cell lines is involved in the regulation of ES cell proliferation (Elzi et al., 2015).

##### 2.3.3.2 EWS-FLI1 protein and transcription

This *EWS-FLI1* fusion gene leads to the formation of the EWS-FLI1 fusion protein, whose activator domains are more potent than those of FLI1 alone as a result of N-terminal domain substitution during chromosomal translocation (May et al., 1993). This property enables EWS-FLI1 to act as an aberrant transcription factor, thus activating the expression of numerous oncogenes. After translocation into the cell nucleus, EWS-FLI1 binds to its consensus sequences located at the promoters of its target genes. Using chromatin immunoprecipitation experiments, several studies have identified the DNA-binding motif of EWS-FLI1 *in vitro* and *in vivo* (Gangwal et al., 2008; Guillon et al., 2009; Boeva et al., 2010). EWS-FLI1 is known to bind to the canonical DNA-binding motif of the ETS protein family, the GGAA sequence (Boeva et al., 2010), but it is also capable of binding to GGAA microsatellites, according to the following rule: “the greater the number of repeats, the more the target gene will be expressed”, within the limit of 20 GGAA repeats (Guillon et al., 2009; Johnson et al., 2017) (Figure 3B). In this context, a 2014 study demonstrates that the regulation of target genes by EWS-FLI1 depends closely on the number of repeats of the GGAA motif at the DNA-binding domain: genes activated by EWS-FLI1 are associated with GGAA microsatellites (motif repeated at least 4 times), while genes repressed by EWS-FLI1 are associated with the canonical DNA-binding motif of the ETS protein family (1 GGAA sequence) (Riggi et al., 2014).

As this ability to bind to GGAA microsatellites is not observed for FLI1 alone, this confers on EWS-FLI1 specific functions enabling the activation of *de novo* enhancers through the recruitment of BAF (BRG1-or BRM-Associated Factors), a chromatin remodeling complex (Boulay et al., 2017). This modulates the expression of numerous target genes, such as *LOXHD1* (Deng et al., 2022), or activates the expression of genes not expressed in healthy tissues or other tumor tissues outside ES (Vibert et al., 2022).

##### 2.3.3.3 Regulation of EWS-FLI1 expression

EWS-FLI1 expression can be regulated at transcriptional, translational and post-translational levels, and *via* protein-protein interactions (Yu et al., 2023).

At the transcriptional level, EWS-FLI1 expression is promoted by methylation or acetylation of histone 3 (H3K4me3, H3K9ac or H3K27ac) (Montoya et al., 2020) and by the binding of the transcription factor SP1 (Specificity Protein 1) to its promoter. Conversely, expression of miR-145 inhibits *EWS-FLI1* transcription (Ban et al., 2011).

EWS-FLI1 translation is modulated by compounds such as Lovastatin or Tunicamycin, which reduce the protein level of EWS-FLI1, thus reducing primary tumor growth (Wang et al., 1999; Herrero-Martin et al., 2011), but increasing a migratory phenotype (Franzetti et al., 2017). Numerous post-translational modifications can take place on EWS-FLI1, resulting in overexpression or degradation of the fusion protein. For example, phosphorylation of threonine 79 (T79) (Klevernic et al., 2009) or O-GlcNAcylation of EWS-FLI1 (Bachmaier et al., 2009) stimulates the oncogenic function of EWS-FLI1, while its ubiquitination leads to its degradation by the proteasome (Gierisch et al., 2016).

Finally, EWS-FLI1 is known to interact with numerous protein players regulating its transcriptional activity. For example, it can bind with RHA (RNA Helicase A) to increase its transcriptional activity (Toretsky et al., 2006), or with PARP-1 to facilitate transcription (Brenner et al., 2012). Conversely, its interaction with CIMPR (Cation-Independent Mannose 6-Phosphate Receptor) leads to its degradation *via* a lysosome-dependent pathway (Elzi et al., 2015).

##### 2.3.3.4 EWS-FLI1 and ES tumor development

Through its function as a transcription factor, EWS-FLI1 regulates the expression of numerous oncogenes involved in many key tumorigenesis processes (cell proliferation, migration, apoptosis, *etc.*). In particular, EWS-FLI1 has been shown to regulate the expression of numerous transcription factors (Cidre-Aranaz and Alonso, 2015). For example, EWS-FLI1 stimulates the expression of *c-Myc* (Dauphinot et al., 2001), *Gli1* (Glioma-associated Oncogene Homolog 1) (Merchant et al., 2009; Mullard et al., 2020) or *NR0B1* (Nuclear Receptor subfamily 0 group B member 1) (Kinsey et al., 2006; Gangwal et al., 2008), genes involved in tumor progression and metastatic development. Conversely, EWS-FLI1 reduces the expression of genes involved in tumor suppressor mechanism (ex: apoptosis), such as *FOXO1* (Forkhead Box O1) (Yang et al., 2010), or *IER3* (Immediate Early Response 3) (Tsafou et al., 2018), tumor suppressor genes regulating mechanisms such as DNA repair, cell cycle arrest and apoptosis.

By recruiting the BAF complex (Boulay et al., 2017), EWS-FLI1 plays a major role in chromatin accessibility at the promoter and enhancer regions of various genes (Tolstorukov et al., 2013) and its role in regulating chromatin remodeling is diverted, like p300 (Riggi et al., 2014), towards aberrant transcription of oncogenes. The GGAA microsatellites therefore appear to be a potential therapeutic target for Ewing sarcoma. Their epigenetic silencing (H3K9me3 labelling) inhibited EWS-FLI1 binding to the *SOX2* enhancer (SRY-box transcription factor 2), thereby reducing its expression and altering tumor growth *in vivo* (Boulay et al., 2018). More recently, EWS-FLI1 has been shown to be directly involved in changes in chromatin configuration (Showpnil et al., 2022). Indeed, through its binding to DNA, *via* GGAA microsatellites, EWS-FLI1 plays a major role in reprogramming the 3D structure of chromatin, by disrupting, for example, TADs (Topological Associated Domains, highly self-interacting genomic regions, delimited by regions enriched in CCCTC binding factor (CTCF) and involved in the regulation of gene expression by limiting enhancer-promoter interactions within the same TAD (Dixon et al., 2012)), or by modulating chromatin loops, with the direct consequence of altering transcription in ES (Showpnil et al., 2022).

Finally, EWS-FLI1 is also involved in ES tumorigenesis by inducing genomic instability. Indeed, it has been shown that EWS-FLI1-induced transcriptional regulation promotes an accumulation of R-loops (Gorthi et al., 2018). These triple-stranded nucleic acid structures (DNA-RNA complex associated with a single-stranded DNA molecule) are rare products of transcription, with a particular conformation and influencing many cellular processes (Aguilera and García-Muse, 2012). In ES, these structures sequester the *BRCA1* (Breast cancer type 1 susceptibility) gene, preventing its expression, and its crucial role in the response to DNA damage (Gorthi et al., 2018).

## 3 Targeted therapies

The lack of progress in chemotherapy-based treatments, particularly for patients with poor response or metastatic disease at diagnosis, has led to the development of new targeted therapies.

By way of illustration, various key cellular processes in tumor development such as DNA repair, with inhibitors of Poly (ADP-Ribose) Polymerase 1 (PARP1), or the cell cycle, with inhibitors of Cyclin-Dependent Kinase (CDK) have been or are currently being studied (Supplementary Table S2). For example, an initial trial showed that the use of a PARP1 inhibitor, Olaparib, alone had no beneficial effect on ES patients (Choy et al., 2014). However, preclinical studies suggesting the benefit of this treatment in combination with other therapies such as Temozolomide, Irinotecan or Ceralasertib led to the initiation of phase I/II clinical trials (ClinicalTrials.gov No. NCT01858168, ClinicalTrials.gov No. NCT02044120 and ClinicalTrials.gov No. NCT02813135) combining Olaparib or Niraparib, with Temozolomide, Irinotecan and Ceralasertib. With regard to cell-cycle targeting, it has been shown, for example, that ES cells require CDK4 and Cyclin D1 to survive and grow independently of anchoring (Kennedy et al., 2015). Thus, a clinical trial targeting CDK4/CDK6 is still underway (ClinicalTrials.gov No. NCT02644460) to study the tolerated dose of Abemaciclib, an inhibitor of these kinases involved in cell-cycle regulation, and in particular in G1 phase regulation. A clinical trial was also initiated last year (ClinicalTrials.gov No. NCT05275426) to determine whether LY2880070, a Checkpoint kinase 1 (Chk1) inhibitor capable of regulating the G2/M transition of the cell cycle in combination with the chemotherapeutic agent gemcitabine could be an effective treatment for ES (Supplementary Table S2). Another approach being considered is the use of CAR-T cells. Indeed, there are clinical trials underway using this method to treat pediatric solid tumors (Supplementary Table S3). Different targets are under study, such as B7-H3, or other surface antigens. If this line of action proves its safety and its anti-tumor activity depending on the target, the use of CAR-T cells could be considered as a promising approach to treat ES.

Because EWS-FLI1 i) is characteristic of 85% of ES cases, ii) regulates numerous oncogenes, and iii) that development of ES depends on this fusion protein, targeting EWS-FLI1 appears as an interesting approach in new therapies for ES. This section therefore focuses on approaches to target EWS-FLI1 that have led to clinical trials, by targeting its interactome or using RNA interference (see (Flores and Grohar, 2021)). There is currently a phase II clinical trial aiming to target EWS-FLI1, by using TK216, an analog of YK-4–279 (Clinical trial.gov No NCT05046314), in combination with vincristine in patients with relapse or treatment-refractory ES. This clinical trial started after a first phase I clinical trial having demonstrated the non-toxicity of TK216 (Clinical trial.gov No NCT02657005). YK-4–279 inhibits the interaction between EWS-FLI1 and RNA helicase A, which is known to enhance EWS-FLI1 activity (Toretsky et al., 2006), and is also able to induce G2/M cell cycle arrest and apoptosis in synergy with vincristine (a microtubule destabilizing chemotherapeutic agent) (Zöllner et al., 2017). However, TK216 has recently been shown to exhibit toxicity to many cancer cell lines by targeting microtubules (Povedano et al., 2022), rather than directly targeting EWS-FLI1 in ES cells, so further study should be considered for the current clinical trial. Another clinical trial is currently in progress, using the RNA interference method (Clinical trial.gov No NCT02736565). Indeed, pre-clinical studies have shown the interest of silencing EWS-FLI1 *via* RNA interference approaches (Chansky et al., 2004; Prieur et al., 2004). The current phase I clinical trial aims to study the safety and the toxicity to target EWS-FLI1 by a lipoplex shRNA. However, the short half-live of this shRNA would require a repeated administration, thus limiting their suitability for use in clinic. The limited number of clinical trials targeting EWS-FLI1 may be explained by the challenges of targeting this fusion protein. Indeed, EWS-FLI1 is a transcription factor with no enzymatic activity.

## 4 Conclusion

A better understanding of the cellular and molecular mechanisms governing ES development has led to the emergence of targeted therapies, such as those targeting EWS-FLI1. In this context, new pre-clinical approaches are currently being studied, involving the targeting of EWS-FLI1 expression, epigenetic changes induced by this fusion protein, or the regulation of the EWS-FLI1 target expression. It has been shown that Proteolysis Targeting Chimeric Molecules (PROTACs) could be an interesting method for targeting EWS-FLI1 degradation. Indeed, one study demonstrated that polyubiquitination of EWS-FLI1 to Lysine 380 led to its degradation (Gierisch et al., 2016). PROTACs are bi-functional compounds that induce proteasomal degradation of targeted proteins by recruiting both the target protein and an E3 ubiquitin ligase (Paiva and Crews, 2019). Although this Lysine 380 is also found in several members of the ETS family, the design of a PROTAC targeting this motif could be a promising approach because of the short-life of EWS-FLI1 (Gierisch et al., 2016). Even if there is currently no clinical trial using PROTACs targeting EWS-FLI1, this innovative technology is developing rapidly in preclinical studies. One such study using PROTACs targeting BET proteins in ES cells showed the potential interest of this approach, by reducing their proliferation and inducing apoptosis in these ES cells. Consequently, targeting EWS-FLI1 seems to be of great therapeutic interest in the treatment of ES, as this fusion protein is necessary for the development of this tumor.
