# Supplementary material for: Ewing sarcoma from molecular biology to the clinic
Source: Front Cell Dev Biol. 2023 Sep 11;11:1248753. doi: 10.3389/fcell.2023.1248753 (PMC10518617; doi:10.3389/fcell.2023.1248753)
Supplement: Supplementary file 1 [file Table1.docx]

| **Clinical trial** | **Phase** | **Drug** | **Status** | **Localization** | **Opening date** | **Closing date** |
| --- | --- | --- | --- | --- | --- | --- |
| NCT02013336 | I | Liposomal Irinotecan - Cyclophosphamide | Recruiting | US | December 2013 | December 2023 |
| NCT02536183 | I | Liposomal Doxorubicin | Recruiting | US | October 2016 |  |
| NCT03507491 | I | Gemcitabin – Nab-paclitaxel | Recruiting | US | August 2018 | December 2023 |
| NCT02611024 | I /II | Lurbinectedin - Irinotecan | Recruiting | Spain, US | May 2016 | November 2023 |
| NCT04067115 | I / II | Trabectedin 1MG - Irinotecan | Recruiting | US | January 2021 | June 2024 |
| NCT05734066 | I / II | Lurbinectedin | Recruiting | US | May 2023 | December 2026 |
| NCT04901702 | I / II | Liposomal Irinotecan with Talazoparib or Temozolomide | Recruiting | Canada, US | June 2021 | December 2025 |
| NCT002945800 | II | Nab-Paclitaxel – Gemcitabine | Recruiting | US | October 2016 | December 2023 |
| NCT04791228 | II | Liposomal Doxorubicin | Recruiting | US | March 2023 | December 2024 |
| NCT05131386 | II | Trabectedin | Recruiting | Spain | May 2021 | July 2024 |
| NCT03359005 | II | Vincristine -Irinotecan - Temozolomide | Recruiting | China | February 2018 |  |
| NCT01864109 | II | Cyclophosphamide – Doxorubicin – Vincristine – Ifosfamide – Etoposide – Temozolomide – Irinotecan – Mesna – Dexrazoxane – G-CSF | Recruiting | US | May 2013 | May 2027 |

**Supplementary Table S1** : Ongoing chemotherapy clinical trials.

**Supplementary Table S2** : Ongoing clinical trials targeting PARP and cycle effectors.

| **Target** | **Clinical trial** | **Phase** | **Drug** | **Status** | **Localization** | **Opening date** | **Closing date** |
| --- | --- | --- | --- | --- | --- | --- | --- |
| **PARP** | NCT02813135 | I/II | Olaparib and Irinotecan or Ceralasertib | Recruiting | Denmark, France, Italy, Netherlands, Spain, United Kingdom | August 2016 | August 2027 |
| **Cdk4/6** | NCT02644460 | I | Abemaciclib | Recruiting | US | February 2016 | December 2024 |
|  | NCT03709680 | I /II | Palbociclib and Irinotecan and Temozolomide and/or Topotecan and Cyclophosphamide | Recruiting | Belgium, Brazil, Bulgaria, Canada, Czechia, France, Germany, Hungary, India, Israel, Italy, Korea, Republic of Netherlands, Poland, Slovakia, Spain, Sweden, Turkey, United Kingdom, US | May 2019 | July 2024 |
|  | NCT04238819 | I /II | Abemaciclib and Temozolomide or Temozolomide and Irinotecan or Dinutuximab, GM-CSF, Irinotecan and Temozolomide | Recruiting | Australia, Belgium, Canada, France, Germany, Italy, Japan, Spain, US | November 2020 | June 2027 |
|  | NCT05440786 | II | Abemaciclib, Irinotecan and Temozolomide | Recruiting | Australia, France, Germany, Italy, Japan, Spain, US | September 2022 | February 2027 |
| **Cdk9** | NCT03604783 | I | TP-1287 | Recruiting | US | December 2018 | June 2024 |
| **Chk1** | NCT05275426 | II | LY2880070 - Gemcitabine | Recruiting | US | March 2022 | March 2024 |

**Supplementary Table 3**: Ongoing CAR-T cells clinical trials

| **Drug** | **Clinical trial** | **Phase** | **Target** | **Status** | **Localization** | **Opening date** | **Closing date** |
| --- | --- | --- | --- | --- | --- | --- | --- |
| CAR-T cells | NCT04897321 | I | B7-H3 | Recruiting | US | June 2023 | March 2025 |
|  | NCT04483778 | I | B7-H3 | Active – Not recruiting | US | July 2020 | December 2025 |
|  | NCT03618381 | I | EGFR | Recruiting | US | June 2019 | June 2024 |
|  | NCT03635632 | I | GD2 | Recruiting | US | April 2019 |  |
|  | NCT04433221 | I /II | Surface antigens | Recruiting | China | July 2020 |  |
|  | NCT03356782 | I / II | Surface antigens | Recruiting | China | December 2017 | November 2023 |
